# The Effect of CEO on Bank Efficiency: Evidence From Private Commercial Banks

**DOI:** 10.3389/fpsyg.2021.738210

**Published:** 2021-09-21

**Authors:** Israr Khan, Wang Mansi, Kuen-Lin Lin, Chi-Fang Liu, Kwanrat Suanpong, Athapol Ruangkanjanases

**Affiliations:** ^1^School of Management, Guangzhou University, Guangzhou, China; ^2^Department of Business Administration, Cheng Shiu University, Kaohsiung, Taiwan; ^3^Chulalongkorn Business School, Chulalongkorn University, Bangkok, Thailand

**Keywords:** Corporate Governance, CEO attributes, financial performance, CEO, banking

## Abstract

The main purpose of this study was to analyze the effects of Chief Executive Officer (CEO) Key attributes on the financial performance of banks. Current literature gives little attention to the important characteristics of CEOs, therefore, this paper investigates the effects of characteristics of CEOs, such as education, experience, nationality, military background (MTB), and political connectedness (PC), on the financial (return on assets) performance of listed private commercial banks in Pakistan. This research sample included 20 private commercial banks of Pakistan and used Secondary data that was derived from 2011 to 2020, which contained 200 sample observations. This paper used the Fixed effect model, Normality test, Breush–Pagan, white test, multi-collinearity, and Augmented Dickey–Fuller test to investigate the study hypotheses. The main results revealed that CEO MTB and PC significantly and positively affected the financial performance of the bank. It is also found that the CEO's education and Experience have a significant and positive relationships with bank profitability. In contrast, the nationality of the CEO has no significant relationship with the financial performance of the bank.

## Introduction

Financial and economic crises in Asian countries and few organizational scandals turn the concentration of researchers toward Corporate Governance (CG). The performance of organizations depends on their governance, and an economy is financially stable when all the enterprises in that economy are better organized and have a good CG. Many organizations fail due to poor CG because CG attracts the concentration of investors and controllers. An efficient and effective regime becomes a primary variable to contend with and endure in the world of competition. Good CG practices assure the achievements of individual firms and strengthen economic advancement (Vinten, 2000). Therefore, economies are engaged in advancing high-quality CG and making different approaches and strategies to manage the importance of governance structure. Previous studies (Claessens et al., 2002) add the identification and solution of different issues related to CG. As suggested by Shleifer and Vishny (1997), governance mechanisms become more complicated with technological advancements, and globalization also creates more complexity in CG, increases business size, and needs more intermediaries, creating more problems (Fernandes and Marlius, 2018). Keep in view the CG issues and the requirement for regulations, various countries issue their standards of governance as per their conditions and requirements. At the end of the economic crisis in 1998, every country gives special attention to CG. They presented different authorities to control the corporate area with the World Bank (WB) assistance and its supervision. Like different states, Pakistan also focused on the efficiency of the CG in January 1999, the Security and Exchange Commission of Pakistan (SECP) officially began operations the first time. SECP started operation under the umbrella of the SEC Act, 1997, in March 2002; Pakistan initially announced a broad code of CG. The Code incorporated particular amendments in SECP Ordinance, 1969, 1997, and Companies Ordinance, 1984 with the help of different controllers, such as the State Bank of Pakistan (SBP), WB, Asian Development Bank, and Stock trades. Specific revisions related to the Code of CG were made in 2012 and 2013 in the SECP act. For better and efficient control, the Commission is divided into subdivisions, departments, and wings. Other than SECP, different authorities direct and control specific organizations, e.g., the stock exchange directs listed organizations in specific angles. SBP gives direction to the banking organizations and financial institutions. SECP, as a team with the Economic Affairs Division of Pakistan and UNDP, launched a scheme in August 2002 to ensure the Code of CG performance. In 2007, SECP, in a joint effort with the Pakistan Institute of Corporate Governance (PICG) and International Finance Corporation (IFC), led a survey to investigate Pakistan CG. The survey included some financial institutions, locally scheduled firms in the stock market, and several non-scheduled organizations. The survey results show that there is a lack of knowledge among organizations concerning CG. Therefore, to increase the awareness about the benefit of the Code of CG, SECP with IFC, and PICG held some training sessions for the organization's governing body.

Board diversity is a significant part of CG, and much literature exists on the correlation between board variables and the financial performance of a bank, e.g., (Pearce and Zahra, 1992; Bhagat and Black, 2001; Adams et al., 2010; Khan and Wang, 2021). However, there is no research about the relationship between Chief Executive Officer (CEO) characteristics and the financial performance of the bank (Nicholson and Kiel, 2007). Jackling and Johl (2009) found that the relationship between the characteristics of a CEO and the financial performance of the bank is vital in understanding CG practices. This investigation aims to explore the effect of CEO characteristics on the financial performance of the private commercial bank. Those people who run the firms choose their objective, e.g., gain or loss. The “CEO” is the most dominant and forceful among the people who run the organization. Being a pilot of an Airplane, the CEO is responsible for improving and diminishing the financial performance of the bank. CEO does a vital job in a bank (Hambrick and Mason, 1984; Rahman et al., 2017), especially in choosing whether a corporation remains in a present market or changes to other targeted markets (Stoller, 2009). In this way, “swimming” or dropping of an organization relies upon CEOs (Westerberg et al., 1997). It is reasonable to concentrate on CEOs as an investigation subject (Rahman et al., 2017). Numerous investigations have shown that the relationship between CEO military background (MTB) and Corporate performance. Sunder et al. (2017) explore that patent declarations by MTB CEOs boost abnormal returns below those by non-military CEOs. Additionally, Benmelech and Frydman (2015) find a negative impact of MTB CEO on corporate performance. You et al. (2020) discover a lower yield on sales when the CEOs are with MTB. Most likely as conformity, regulation, and bureaucratic conduct, which are encouraged in military service; debilitate entrepreneurial conduct and innovativeness (Avrahami and Lerner, 2003) in this way yielding and lower incomes than firms led by non-MTB CEOs. The relationship between politics and trade has been clarified by the advantages produced for both politicians and traders. As indicated by Brogaard et al. (2015), political power in a corporation enables its executives and directors to influence laws and regulations. It gives them admittance to internal information, which empowers them to imagine economic changes and decrease uncertainty. Earlier studies have analyzed political connectedness (PC) effects on the performance of the corporation (Gilabert, 2011; Li et al., 2015). Ling Zhou examines the impact of firms' PC on outside financing, company investment, and economic performance (Ling et al., 2016); the findings of the examination show that political relationships are negatively identified with ROA (Return On Asset). Politically connected directors negatively affect the bank performance. Hung et al. (2017) found a positive effect on the bank performance (AlQudah et al., 2019).

Prior investigations have found the importance of the education of a CEO on the bank performance (Darmadi, 2013b; Phan, 2016), which show that the education level influences the decision-making ability of a CEO. As per Hambrick and Mason (1984), education indicates a CEO's knowledge, intellectual, and other human resource abilities. Darmadi (2013b) proves that the educational qualifications of a CEO decide productivity and bank value. As the education level increases, a CEO becomes more engaged and had practical experience in their field. Gupta and Mahakud (2020) show that the education of CEO increases bank performance. Elsharkawy et al. (2018) found a positively insignificant relationship between CEO's education and bank performance.

Experience of the CEO is needed in the classic business domains (e.g., financial, advertising, operations, or HR). Hambrick and Mason (1984) arranged practical experience dependent on whether it is oriented toward yield, throughput (financial and production), or peripheral behavior (law, finance, and HR) (Datta and Rajagopalan, 1998; Barker and Mueller, 2002). Experience may give valuable, relevant knowledge and social relationships; however, it may also secure a CEO in a specific way of understanding and interpreting problems. It could be significant in stable economic conditions; however, it is less important for CEOs of banks to confront financial discontinuities (Hambrick and Mason, 1984). Earlier studies show experience of a CEO is positively connected with corporate performance (Wang et al., 2016; Chou and Chan, 2018). Appointment of foreign CEOs upgrades the value of the decision-making in bank board (Ibrahim and Hanefah, 2016) because the overseas CEOs bring diverse knowledge and perspectives, for example, language, job experiences, customs, behaviors, and rules of the economies, which strengthening the decision-making process (Ruigrok et al., 2007; Ibrahim and Hanefah, 2016). Upper echelon theory contends that the presence of foreign CEOs helps the organization obtain and allocates assets that are critical to the organization's prosperity. Pfeffer and Salancik (2003) show that the foreign CEOs can bring new experiences and diverse ideas to the bank to manage foreign issues. This study uses the performance measurement ROA of an organization. ROA is the proportion used to determine the management capacity of an organization in getting profit by using the total assets owned (Attar and Islahuddin, 2014). ROA measures how successfully an organization can convert revenue from the return on investment into assets. The higher the ROA of an organization, the better the performance of the bank. As per our approach, there is no study on this relationship in Pakistani private commercial banks. Therefore, the motive of this investigation is to spot the impact of the CEO on financial performance regarding Pakistani private commercial banks. Additionally, with the developed research model, the study tends to the accompanying research question:

**RQ:** what attributes of CEOs could affect the private commercial bank's performance?

This research contributes to the literature in numerous ways. We do not get any broad study in the literature that takes such like CEO attributes at, e.g., the effect of CEO on bank profitability of private commercial banks in Pakistan. We include the exogenous literature changes in CG by concentrating on crisis-initiated regulatory changes in the banking area. Our sample covers the post-crisis, large sample size, and a broader era of the time difference to explore the effect of a CEO on bank profitability under normal and in crisis financial conditions. Past studies focused on board size (Bsize), board meetings, and other board structure variables. Still, the present research incorporates CEO attributes accordingly, from which expected to give new knowledge. This study concentrated on all the private commercial banks listed in the SECP, expected to give an overall image of the private investment in the banking industry. A lot of work has been done on the CEO characteristic, but no one can use such a variables combination, especially in Pakistan. In 2002, an objective was assigned to SECP for the background of good CG structure for all corporations in Pakistan, whereby CEOs monitor and control devices under best CG practices to accomplish the goals of investors. When these reforms were implemented in the corporate sector, its performance increased (Mir and Nishat, 2004). In this implementation period, performance evaluation research studies are considered to be performed from time to time to examine its effect. Some investigations have already been conducted in this sector, but their research focuses on different variables, and they have used different data analysis techniques and models. However, there is still a need to perform more investigations in this sector with increases in data sample size and further developed analytical methods to explore whether the different CEO characteristics significantly affect bank performance. The objective of this investigation is to explore this issue in the Pakistani context. Pakistan as an emerging economy gives a stimulating context to investigate the effect of CEO's attributes on bank performance because of some dependably unusual social elements. For example, Pakistan is identified as a state dependent on ethnic affiliations. The investors suffer from a general circumstance known as favoritism or “Wasta.” These wonders put the focus of investors and decision- makers to select CEOs based on relationships, for example, family, clan, kinship, and companionship regardless of attributes, such as, capabilities, experience, education level, and health to perform the work (Omran et al., 2008), which may adversely affect the viability of the management and on translucent financial exposure. We analyze all of these variables to acknowledge the fundamental contributions to this study and plan research gaps. This investigation aims to add to this meager literature by investigating CEO political ties' effect on banks' performance. Pakistan is a fascinating case for investigating this association for a few reasons. First, CEO's political ties typically impact on corporate sector of Pakistan (Khwaja and Mian, 2005; Saeed et al., 2014). Second, the development of financial markets, insufficient institutional help, overall legislative control, and interference generate business obstacles. The continuation of this paper is sorted out as follows.

In section “literature review and hypotheses development”, we described the literature review and hypotheses development. The section “theoretical perspectives” shows the methodology and definition of variables. Section “Methodology” comprises results and discussion. The section “Results and Discussion” describes the conclusion and policy implication of this research.

## Literature Review and Hypotheses Development

### CEO With an MTB and Bank Performance

Military service can change the behavior of people in different manners that could influence behavior and decision after they become CEOs in the future (Benmelech and Frydman, 2015). In contrast, psychological literature shows that MTB CEOs are related to boldness, aggressiveness, and adventuresome behavior (Elder, 1986). A couple of ongoing studies found that organizations operating by CEOs with MTB are less inclined to perform better (Benmelech and Frydman, 2015; You et al., 2020). For instance, Benmelech and Frydman (2015) find that CEOs with an MTB put less in Research and Development (R&D) and follow less organization performance. Continuously, You et al. (2020) show that organizations led by military CEOs are more likely to make small R&D expenditures than their non-MTB counterparts in China. This proof can be clarified by the logic that military training and service values subordination to political authority, obligation, commitment, and kindness, prompting corporate literature that inspires little risk-taking and conservative investment behavior (Franke, 2001; Benmelech and Frydman, 2015). Therefore, we hypotheses that:

**H1:** A CEO with an MTB in private commercial banks is significantly and negatively related to bank performance.

### Political Connected CEO and Bank Performance

However, when an organization cannot use the available resources efficiently, it may negatively affect organizational performance. Political intervention of a CEO in the organization and a weak administrative ability of politicians can be decrease organizational performance (Boubakri et al., 2012). For example, Asquer and Calderoni (2011) found a negative impact of political associations on Italian corporation performance. Similarly, Bertrand et al. (2007) show that organizational and political connection prompts a negative impact on ROA. A study by Faccio (2010) indicates that politically connected organizations have lower performance than non-political connected firms. Crook et al. (2011) found that the effect of Politically connected CEOs (PCCEOs) is low investment effectiveness and organizational performance. Furthermore, Boubakri et al. (2008) indicate that politically connected organizations show a weak financial performance compared to their non-connected counterparts. Finally, Disli et al. (2013) found that politically associated CEOs in Turkish corporations slow down investors' motivating monitor and control of their banks. Briefly, it concluded that politically connected CEOs might decline bank performance. Therefore, we suggest the following hypothesis:

**H2:** Politically connected CEO in private commercial banks is significantly and negatively related to bank performance.

### CEO Level of Education and Bank Performance

Human capital comprises the arrangement of abilities and information obtained through education and experiences that empower workers to play out their obligations, which deliver their economic values (Jansen et al., 2013). Therefore, the education level of the CEOs is vital for the corporation as it influences performance and decides its gain or loss (Pennings et al., 1998). The personnel of a firm is also called intangible assets that an organization cannot copy or substitute. They give a base to an organization's competitive advantages (Barney, 1991; Carpenter et al., 2001). Therefore, the education level of a director and training are comprised of their knowledge, aptitudes, and capacities—which include an intangible asset for the corporation (Crook et al., 2011). Individuals with higher formal education levels have more critical information and learn organization-specific knowledge (Hitt et al., 2001). Higher education levels also grow individuals' cognitive difficulty and are connected with innovation (Hambrick and Mason, 1984). Furthermore, education increases the self-confidence of an individual and positively impacts the degree of risk tolerance and the capacity to manage uncertainty (Mitchell et al., 2005; Jansen et al., 2013). The education level is considered a crucial part of upgrading an organization's performance (Makhlouf et al., 2017). Past investigations, e.g., (Carpenter and Westphal, 2001; Darmadi, 2013a), show that the education level of a CEO considers a personal cognitive capacity and abilities. For example, when CEOs have an advanced education level, they obtain good strategic ideas and decision-making skills because this advanced education quit access to original ideas. In addition, executive bodies with an advanced level of education have a more intellectual, scholarly influence, creative thoughts, and distinctive perspectives, all of which enable them to manage various problems effectively. This shows that an increase in education level enhances organization management competence and increases competition in the industry. Therefore, we hypothesize that the higher level of education, the higher the performance of the bank.

**H3:** CEO with a higher level of education in private commercial banks is significantly and positively related to bank performance.

### CEO Experience and Bank Performance

According to the study of Hambrick and Mason (1984), experience means skills, and it is regardless of education. In his evaluation, the effect of the board of directors' education on corporate performance, he also recommends that experience is a more potent variable in the board of director's capabilities. This idea may increase from the realistic experience compared with the conceptual and workbook methodology of formal education. It is found that the experience of an outside director positively affects the financial and market performance of an organization—previous experience of the CEO positively affects the financial performance of a bank in an unstable industry. Experience positively impacts the performance of a bank in a stable industry (Hambrick and Mason, 1984). Specialized experience of a CEO has been found to affect a CEO's activities positively and efficiently in leading organizations (Hambrick and Mason, 1984; Herrmann and Datta, 2006; Crossland et al., 2014). In summing up, CEOs would be empowered to get more knowledge and ability with more experience, which would interpret more tactical decision-making and enhanced bank performance. Therefore, we hypothesize that the higher the experience of the CEO will be higher the performance of the bank.

**H4:** Experience of a CEO in private commercial banks is significantly and positively related to bank performance.

### Foreign CEO and Bank Performance

Chief Executive Officer global experience helps organizations in making international competitiveness through worldwide diversification. Such experience grooms directors for adapting to unexpected problems and new issues. Therefore, global experience has become a requirement for a CEO (Bass and Bass Bernard, 1985; Black, 1999). With increasing globalization, the organization will change the framework of its management by having more foreigners on its top management (Sanders and Carpenter, 1998; Oxelheim et al., 2013). In such a manner, organizations try to draw foreign directors to add managerial abilities and particular skills (Sanda et al., 2008). Suppose that CEOs with different backgrounds, especially nationality, can encourage organizations to understand comprehensive sensitivities, which help them in entering new international markets (Carter et al., 2003; Hillman and Dalziel, 2003; Pfeffer and Salancik, 2003; Abu et al., 2016; Scheppink, 2018). As a result, both CG and corporations prefer to assign directors with overseas nationality or experience (Oxelheim and Randøy, 2003; Masulis et al., 2012; Rose, 2016; Ware, 2016; Rahman, 2018). Past studies found that overseas executives have a positive correlation with the financial performance of an organization (Rosenstein and Wyatt, 1997; Carter et al., 2003; Oxelheim and Randøy, 2003; Müller, 2014) in the Netherland (Overveld, 2012), Nigeria (Abu et al., 2016), Korea (Choi et al., 2007), and Kenya (Karani, 2015). Conversely, it is found that foreign directors have low participation and a feeble monitoring role because of their home abroad. Furthermore, language differences and newness to or little learning of the neighborhood culture, market, and economy also diminish their effectiveness. Therefore, foreign CEOs have no binding impact on the stock exchange and organization market value in rising economies, such as Turkey, Indonesia, and Pakistan. As a result of the dissimilar and rare literature that concentrates on the developed economies, this study further investigates the relationship between foreign CEO and bank performance in rising countries, such as Pakistan. Additionally, examine the investigation builds up the accompanying hypothesis.

**H5:** Foreign CEO in private commercial banks is significantly and positively related to bank performance.

## Theoretical Perspectives

According to the study of Bathula (2008), three significant theoretical perspectives support the discussion on board attributes and CG systems. However, Upper Echelons Theory (UET) is used in this paper as a framework.

### Upper Echelons Theory

In exploring the effect of CEOs on bank performance, this paper explores the UET, as devised by Hambrick and Mason (1984). This theory describes that managerial background attributes can reflect tactical decisions. The basic principles of UET are derived from leap rationality theory, which expresses that the decision-making of an individual is not entirely based on rational motives because these cannot capture all relevant information about the subject (March, 1978). Consequently, people generally rely upon their emotional and behavioral factors in decision-making (Hambrick and Mason, 1984), which means that the study of executive decision-making must consider the difficulties in measuring the psychological components of CEOs. Researchers work on UET and additionally contended that directors' apparent demographic indicators (for example, education level, business degree, age, gender, ethnicity, political background, and tenure length, etc.) could suggest to their characters; these are objective and measurable (Marcel, 2009; Abdullah and Said, 2018). By drawing on UET, a few studies have revealed that how different attributes of executives, for example, gender (Kassinis et al., 2016), age (Lee et al., 2018), education (Manner, 2010), ethnicity (Louis and Osemeke, 2017), and political belief system (Chen, 2015), can affect the financial performance of a firm. Generally, our UET-based investigation concentrated on noticeable attribute indicators of CEOs, which comprises education, experience, nationality, political association, and MTB, to clarify the level of effect they have on corporate financial performance.

## Methodology

### Research Design

This research uses a quantitative approach because this manuscript aims to investigate the relationship of independent variables, such as military, political connection, education, experience, and nationality of a CEO, with the dependent variable as ROA. The sample of this research includes 20 private commercial banks of Pakistan. Secondary data have been derived from 2011 to 2020. This period covers the post-crisis and the latest data, and the latest data improve bank governance policies. Next, this is a large sample size and a broader era of the time difference to explore the effect of a CEO on bank profitability in different financial conditions. The total number of observations is 200. Data associated with CEO attributes and financial performance (ROA) are collected from the audited published annual reports and balance sheets of the chosen banks, just as from SBP and LinkedIn publications. Political data were collected from the certified webpage Election Commission of Pakistan (ECP), which holds elections for the National and Provincial Assemblies. A few banks were excluded because the data were not extensively accessible.

### Dependent Variable

ROA is a dependent variable that shows bank performance. ROA is known as the income produced by the bank according to its resource base. It is calculated to control the operating performance of a bank (Yim, 2013; Serfling, 2014). This methodology is also implied by Mishra and Nielsen (2000) and Peng et al. (2007). ROA is measured as net profit after tax to total assets by 100.

### Independent Variables

Chief Executive Officer with an MTB: In this research, an MTB of CEO is represented by MTB, a dummy variable that takes one if a CEO is an MTB in a given year and zero if otherwise. Some past investigation has also used these variables (Benmelech and Frydman, 2015; You et al., 2020). CEO PC: A CEO is considered politically connected when he stands in the national or provincial election, held from 2011 to 2020. These measures are used in a few different investigations (Asquer and Calderoni, 2011; Saeed et al., 2014). CEO education: Education level of a CEO, for example, holding a higher degree, considers a vital asset for banks and gives a combination of capabilities, expertise that help in applying the governance rules (Ujunwa, 2012). Therefore, in this investigation, the CEO education level is calculated by the percentage of CEOs holding an advanced degree, such as a Master's or Ph.D. degree. This indicated variable has shown up in some past studies (Bhuiyan et al., 2010; Ujunwa, 2012). CEO experience: CEO experience is a significant independent variable. We can measure it in several years. Some past studies also used the experience as an independent variable (Stimpert et al., 2010; Phan, 2016). Foreign CEO: This variable is measured by the proportion of foreign CEOs on the bank boards. In various past investigations, nationality was used as an independent variable (Darmadi, 2011; Ibrahim and Hanefah, 2016).

### Control Variables

To control the effect of economic conditions, this paper used the following control variables. Bank size (BSZ) is an important control variable because large banks are ideal for increasing their performance by guaranteeing proficient usage of their considerable assets (Cheung et al., 2007). Subsequently, financial investors favor large banks that expand their market worth. Bank size is determined by taking the common logarithm of total assets some past investigations also used (Abdullah, 2005; Murtaza and Azam, 2019). Leverage measure as total obligations divided by total assets (Ilmas et al., 2018; Khan and Wang, 2021). Measurement of each variables are exist in Table 1.

**Table 1:** Definition of key variables.

**Variables**	**words**	**The measurement unit of variable**
Return on assets	ROA	$=\frac{\text{NetProfitafterTax}}{\text{TotalAssets}}\times 100$
CEO military background	MTB	Military background CEO = 1, Non-military background = 0
Political connection	PC	A CEO is considered as connected when he stood in the national or provincial election, held from 2011 to 2020
CEO education	CEOEDU	Percentage of CEO's holding an advanced degree, e.g., Master or PhD
CEO Experience	CEOEXP	Experience in a number of years
Foreigner CEO	CEOFER	Proportion of foreign CEO's on the bank boards
Board size	Bsize	Total number of directors on the board
Leverage	LVR	The ratio of total financial debt to total assets

### The Model

The statistical technique of Eviews was applied in the process of data analysis. To test the study hypothesis, we followed the FE model (Jensen and Murphy, 1990; Hermalin and Weisbach, 2006; Buck et al., 2008; Ausat, 2018; Khan and Wang, 2021). The following is the regression equation of this study:


$$\begin{array}{l} ROA_{it}=\beta_{0}+\beta_{1it}MTB+\beta_{2it\;}PC+\beta_{3it}CEOEDU\\ +\beta_{4it}CEOEXP+\beta_{5it}CEOFER+\beta_{6it}Bsize+\beta_{7it}LVR+\mu_{it}(1.1) \end{array}$$

Where i refers to the cross-section, t is time, and μ_it_ is an error term. β_0_ is the Constant coefficient of regression. B_1_-β_5_ refer to the regression coefficient of independent variables. β_6_ and β_7_ are control variables.

### Estimation Strategy

We know that simple Ordinary Least Squares (OLS) model cannot be a perfect estimator. Every board data model is not viewed as the equivalent of time series and cross-section data since it labels to have pairs of subscripts-i and t—where i shows the unit of investigation and t demonstrates time measurement (Baltagi, 2008). The error term of the Pool model comprises of three mechanisms—individual (μi), time-specific effect (λt), and other disorder (vit).


$$\begin{array}{l} Yit=\alpha it+\beta itX+Uit \end{array}$$

Where Uit = μi + λt + vit μi = cross-section property λt = time-specific property vit = other random errors

Based on these kinds of impacts of pool data, two distinct models are presented to manage cross-sectional effects:

### Fixed Effect Model

The FE model investigates the relationship between predictor and response variables inside an entity (country, individual, and organization). Every organization has its separate attributes that may affect the independent variables. For example, being a male or female could affect the thinking toward a particular problem, or the political structure of a specific state could have a few impacts on business or GDP, or the trade approaches of a corporation may affect its stock price. When using FE, we accept that something within the individual may affect or bias the independent or dependent variables, and we have to control for this. The justification behind the presumption of the relationship between the error term and independent variables of the organization. The FE model eliminates those time-invariant characteristics to assess the net effect of the independent variable on the result variable.

## Results and Discussion

### Descriptive Statistics

Table 2 shows the descriptive statistic of all variables implied in this research. These results are beneficial to explain the individual variables and their contribution. The secondary data are collected from different banks working in Pakistan, which contain 200 sample observations. In this study, the financial performance of the bank measured by ROA is considered a dependent variable, and all other variables are considered independent variables. These measurements describe the shape, location, and variation of each variable included in this study. Most of the regression models need some basic assumptions, and this information provides essential information about these assumptions. In this table, skewness and kurtosis provide information about the distribution of each variable. The majority of the variables do not precisely follow the normal distribution but using the theory of central limit theorem, as we increase the sample size, these variables approach a normal distribution. It can be perceived that the average value of the response variable is 0.01, its minimum and maximum values are 0.04 and −0.07, respectively.

**Table 2:** Descriptive statistics.

	**ROA**	**MTB**	**PC**	**CEOEDU**	**CEOEXP**	**CEOFER**	**BSIZE**	**LV**
Mean	0.01	0.07	0.11	0.65	31.24	0.94	9.05	0.90
Median	0.01	0.00	0.00	1.00	31.00	1.00	9.00	0.92
Maximum	0.04	1.00	1.00	1.00	65.00	1.00	18.00	0.98
Minimum	−0.07	0.00	0.00	0.00	15.00	0.00	4.00	0.24
Std. Dev.	0.01	0.26	0.31	0.48	8.28	0.25	2.05	0.08
Skewness	−2.05	3.37	2.49	−0.63	0.45	−3.53	1.01	−4.29
Kurtosis	10.91	12.36	7.21	1.40	3.77	13.45	5.42	29.91
Probability	0.00	0.00	0.00	0.00	0.00	0.00	0.00	0.00
Sum	1.51	14.00	22.00	130.00	6247.00	187.00	1810.00	179.83
Sum Sq. Dev.	0.04	13.02	19.58	45.50	13643.96	12.16	839.50	1.23
Observations	200	200	200	200	200	200	200	200

Table 3 shows a correlation between predictor and response variables, and a robust measurement implied in assessing the relationship between pair of variables. It has values between −1 and +1, where the positive figure shows that both variables proceed in a similar direction while the negative value shows their opposite direction. It can be observed that MTB, leverage and ROA, CEO education and MTB, leverage and CEO experience, political connection, CEO education, CEO nationality, and CEO experience have a negative correlation. In contrast, return on assets, political connection and Bsize, CEO education, experience, nationality are positively correlated. CEO education and ROA have the highest correlation, and their value of correlation coefficient is 0.01. The weakest correlation is between CEO experience and CEO political connection (*r* = 0.09).

**Table 3:** Correlation analysis.

	**ROA**	**CEOEDU**	**CEOEXP**	**CEOFER**	**MTB**	**PC**	**BSIZE**	**LV**
ROA	1.00							
CEOEDU	0.01	1.00						
CEOEXP	0.03	−0.31	1.00					
CEOFER	0.19	0.02	−0.11	1.00				
MTB	−0.01	0.12	−0.10	−0.01	1.00			
PC	0.04	−0.01	0.09	−0.04	0.15	1.00		
BSIZE	0.05	−0.08	0.08	−0.32	−0.03	0.02	1.00	
LV	−0.18	0.12	−0.26	−0.04	0.04	−0.01	−0.02	1.00

Table 4 shows a complete estimation of formulated hypotheses, their significant values, and all measures used for model specifications. Our findings show that military and non-MTB CEOs differ from one another. Regression analyses show a significant negative relationship between CEO with an MTB and bank financial performance. The beta coefficient and its *P*-values are β = −0.0121, *P* = 0.0012, respectively. When the CEO of a bank has an MTB, the financial performance of a bank is low. The beta coefficient and its *P*-values are significant at the 01% level. Hence, the financial performance of banks led by CEOs with MTB is lower contrasted with those managed by the CEO with a non-MTB. The results also show that a CEO with military experience conditionally has a negative effect on the performance of a bank. The effect of politically connected CEOs on bank performance is shown in Table 4. The coefficient of PC is negatively significant at the 01% level, which shows that banks with politically connected CEOs have lower financial performance than those non-political relations. The results also show that the banks led by politically connected CEOs are underperforming instead of non-political banks. These suggest that PC intensifies agency problems by influencing the management to be busy in selfish activities that secure the interests of PC CEOs, decreasing bank performance. Our results are consistent with the results of the studies by Faccio (2006, 2010), Fan et al. (2008), and Khawaja and Mian (2009), each of whom shows a low performance of PC banks. The results show that the correlation between the CEO level of education and bank performance is significantly positive under the first hypothesis. The beta coefficient and *P*-values are, respectively, β = 0.0060, *P* = 0.0044. This result is related to the required results. These results confirm and accept the first hypothesis **H3** of the study. A significant positive impact suggests that a CEO with a higher education level (Ph. D. or Master Degree) in a board gives more significant bank profitability levels. This study is consistent with past research studies (Mohamed Yunos, 2011; Pulungan and Sadat, 2014). Besides, when a CEO has a higher education level, they acquire good tactical thinking and decision-making skills. Finally, this study guesses that all Pakistani listed private commercial banks must have a qualified CEO and have an advanced education degree. Furthermore, Table 4 shows that the beta coefficient and *P*-values of experience and education are, respectively, β = 0.0001, *P* = 0.00372 and β = 0.0060, *P* = 0.0044); experience values is less than education level. Even though both descriptive variables are positively and statistically significant, but education is stronger in deciding ROA. Therefore, we can accept the fourth hypothesis, **H4**. However, it cannot support the recommendation of Phan (2016), and the reason is that experience is a strong variable than education for management capabilities. Formal education is required for the development of information, aptitude, and attitude. Regression analysis in Table 4 shows that foreign CEOs (CEON) have an insignificant relationship (β = 0.0141, *P* = 0.3405) with bank performance. However, according to the past literature, the foreign experience of a CEO contributes to the management abilities and other specialized talents, which guarantees the efficiency and expertise lead of the organizations, which improves financial performance (Black, 1999; Carpenter et al., 2000; Daily et al., 2000; Sanda, 2011). Nevertheless, our findings cannot support the fifth hypothesis, **H5**. Therefore, we can reject the fifth hypothesis, **H5**; it means that the nationality of the CEO has no relationship with the financial performance of a bank. However, our findings are consistent with the findings of Arioglu and Borak (2015) and Vania and Supatmi (2014). Nevertheless, the rejection might be because of contextual dissimilarities as the investigations are led in Turkey and Indonesia. The control variable Bsize is significantly positive at the 0.1% level; it means that the large Bsize of a bank has more assets, board members, and market strength to show higher performance. Next, bank leverage shows a significant negative impact on bank performance, representing that positive leverage exercise negatively affects bank performance.

**Table 4:** Regression analysis.

**Constructs**	**Coefficient**	**Std. error**	***t*-statistics**	**Probability**
C	−0.002049	0.014732	−0.139065	0.8895
MTB	−0.012183	0.003712	−3.282453	0.0012^***^
PC	−0.003106	0.003010	−1.031923	0.0334^***^
CEOEDU	0.005925	0.002058	2.878555	0.0044^***^
CEOEXP	0.000184	0.000123	1.492602	0.0032^***^
CEOFER	0.014144	0.003993	3.542240	0.3405
BSIZE	0.001251	0.000479	2.614037	0.0097^***^
LVR	−0.026711	0.012268	−2.177278	0.0307^***^
*R* ^2^	0.5052	Mean dependent variable		0.0076
Adjusted *R*^2^	0.4373	SD dependent variable		0.0141
SE of regression	0.0106	Akaike info criterion		−6.1395
Sum squared Resid	0.0197	Schwarz criterion		−5.7272
Log likelihood	638.9486	Hannan-Quinn criteria		−5.9726
F-statistic	7.4445	Durbin-Watson stat		1.1901
Probability (F-statistic)	0.0000			

*, **, *** *Indicates significance at the 10, 5, 1% levels*.

*MTB, Military background; PC, Political Connectedness; CEOEDU, CEO Education; CEOEXP, Experience; CEOFER, CEO Foriegner; Bsize, board size; LVR, leverage; ROA, Return on Assets*.

The negative effect could be attributed to the high borrowing costs of the Pakistani markets. In regression analysis, *R*^2^ is used for the model's goodness, but statisticians preferred adjusted *R*^2^, which is 43.73% in multiple regressions. To measure the overall significance of the model, the F-statistic is used. The F-statistic *P*-value is 0.000, demonstrating that the overall model is highly significant and well-fitted. The adjusted *R*^2^ value of 0.43 in the regression model shows that 43% of the reported variability in ROA can be described by the differences in variables to be specific CEO attributes. The remaining 57% is not explaining, implying that the remaining 57% of the ROA shift is associated with variables not represented in this model. The results of this paper are not free from a small number of limitations. We derived (secondary) data of private commercial banks for this research, from 2011 to 2020, that restricted our model size to ensure data accessibility. We used 20 private commercial banks in Pakistan. If other banks have chosen, then the consequences should be more attractive. The data relating to just a single country and banking sector of the economy, the relationship between CG and financial performance, can be investigated by taking multiple countries' data and including other significant variables of the CEO's attributes and ownership structures.

### Heteroscedasticity

When the size of the error term contrasts across values of independent variables, then heteroscedasticity is present in the data.

**Figure 1:**
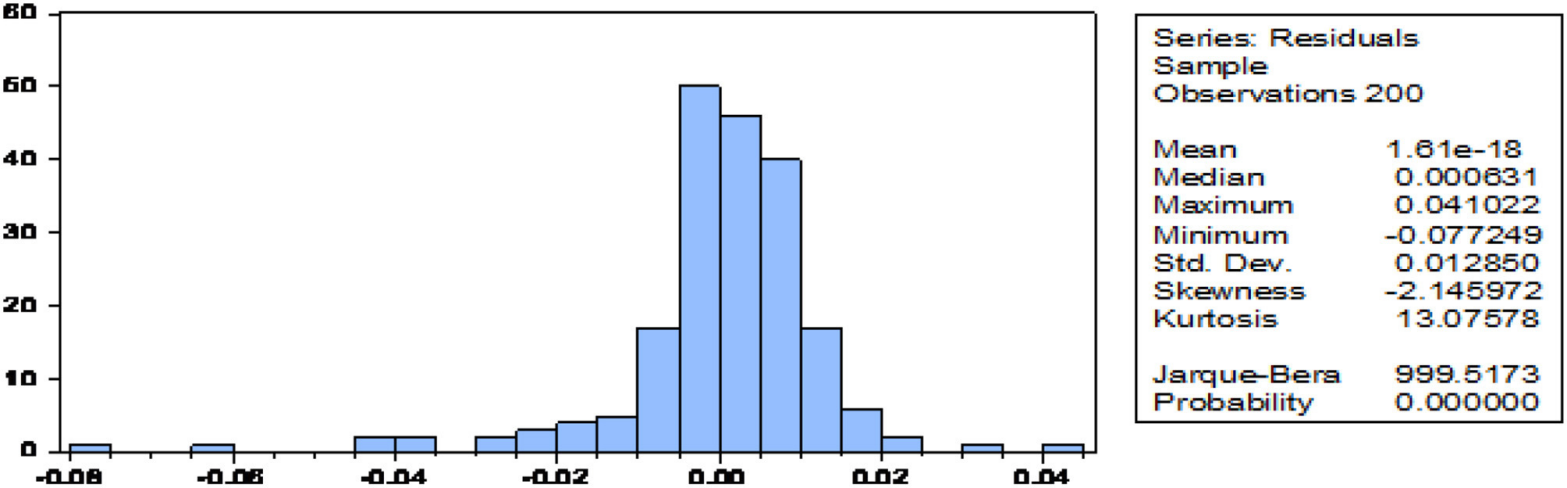
Graphical method.

The normality test results in Figure 1 show that all variables for the study were consistently distributed as exposed by the all probability in the histogram are smaller than the significance values of 0.05.

Table 5 exhibits that the F-statistic value is 35, and Prob (F-stat) is 60%, which is more than 10%, so there is no heteroscedasticity.

**Table 5:** Breush-Pagan test.

**Variable**	**Coefficient**	**Std. error**	***t*-statistic**	**Prob**.
Dependent Variable: RESID^∧^2				
Method: Least squares				
C	0.0016	0.0043	2.1106	0.0355
CEOEDU	−8.8505	8.9805	−0.9823	0.3254
CEOEXP	−1.0105	5.3806	−1.8778	0.0620
CEOFER	−0.0028	0.0074	−0.7377	0.4623
MTB	−1.4105	0.0062	−0.0890	0.9308
PC	−5.4505	0.0031	−0.4191	0.6785
LV	−0.0050	0.0035	−0.6502	0.5134
BSIZE	−4.2205	2.0905	−2.0215	0.0446
*R* ^2^	0.041029	Mean dependent var		0.000164
Adjusted *R*^2^	0.156067	S.D. dependent var		0.000572
SE of regression	0.000571	Akaike info criterion		−12.06042
Sum squared resid	6.25E-05	Schwarz criterion		−11.92848
Log likelihood	1214.042	Hannan-Quinn criter.		−12.00702
F-statistic	1.353529	Durbin-Watson stat		1.594052
Prob (F-statistic)	0.609742			

Table 6 shows that the probability value (F-statistic) is 69%, which is more than 10%; therefore, we can say no heteroscedasticity is present.

**Table 6:** White tests.

F-statistic	0.850601	Prob. *F*_(31,168)_	0.6946
Obs^*^R^2^	27.13259	Prob. Chi-Square (31)	0.6655
Scaled explained SS	150.9798	Prob. Chi-Square (31)	0.0000

Table 7 shows that the values of Variance inflation factor (VIF) are <10%, so we can say that there is no multi-collinearity among the variables.

**Table 7:** Multi-collinearity.

	**Coefficient**	**Centered**
**Variable**	**Variance**	**VIF**
**Variance inflation factors**		
MCEOEDU	4.2406	1.126163
MCEOEXP	1.5208	1.211306
MCEOFER	1.5905	1.132390
MTB	1.3805	1.147951
PC	9.1606	1.136456
LV	0.1451	1.180726
BSIZE	2.2907	1.123439

Augmented Dickey-Fuller test (test for data Stationery)

The fact that Prob(F-statistic) is <0.05 indicates that these study variables are stationary, as shown in Table 8. These results demonstrate that the data in this analysis are stationary and cannot deviate from data stationery assumptions.

**Table 8:** Augmented Dickey-Fuller tests.

Null Hypothesis: ROA has a unit root				
Exogenous: Constant				
Lag length: 4 (Automatic - based on AIC, maxlag = 5)				
			***t*-statistic**	**Prob.** ^*^
**Augmented Dickey-Fuller test statistic**			−5.643228	0.0000
Test critical values:	1% level		−3.463924	
	5% level		−2.876200	
	10% level		−2.574663	
^*^MacKinnon (1996) one-sided *p*-values.				
**Augmented Dickey-Fuller Test Equation**				
Dependent Variable: D(ROA)				
Method: Least squares				
**Variable**	**Coefficient**	**Std**. ***e*****rror**	***t*** **-statistic**	**Prob**.
ROA (-1)	−0.444227	0.078719	−5.643228	0.0000
D [ROA(-1)]	0.030810	0.086393	0.356626	0.7218
D [ROA(-2)]	0.056119	0.081947	0.684816	0.4943
D [ROA(-3)]	0.046523	0.076421	0.608773	0.5434
D [ROA(-4)]	0.199967	0.070154	2.850412	0.0049
*R* ^2^	0.235539	Mean dependent var		8.05E-05
Adjusted *R*^2^	0.215315	S.D. dependent var		0.012444
SE of regression	0.011024	Akaike info criterion		−6.147267
Sum squared resid	0.022967	Schwarz criterion		−6.046560
Log likelihood	605.3586	Hannan-Quinn criter.		−6.106492
F-statistic	11.64658	Durbin-Watson stat		1.987048
Prob (F-statistic)	0.000000			

## Conclusion and Policy Implications

Board is one of the significant components of CG. CEOs are responsible for the overall bank performance to ensure the interest of the shareholders and stakeholders. This paper examines the effect of CEO attributes on the financial performance of private commercial banks regarding a rising and developing economy like Pakistan. This investigation finds a significant and negative relationship between CEO MTB and politically connected CEOs with the financial performance of private commercial banks, which raises questions on the role of CEO MTB and politically connected CEOs on the board of Pakistani banks. The presence of such CEOs in a board becomes at risk, and there should take place an occurrence of the poor financial performance of a bank. These findings show consistency with the past research that if a CEO with an MTB and politically connected CEOs working on top of the bank boards, it will negatively affect the investors and lead the bank performance toward a decline in Pakistan. The findings set up a new perspective for Pakistani investors to look at CG practices of banks while making investment decisions. This investigation results reveal that the negative effect of MTB and politically connected CEO states that there is a need to have transparency in the appointments of CEOs. The sample result also finds a significant and positive relationship between the education level of a CEO and experience with the financial performance of private commercial banks. This study recommends that Pakistani private commercial banks consider the appointment of experienced and well-qualified CEOs in the future. In contrast, Foreign CEO does not show any significant relationship with bank financial performance. Based on the insignificant relationship with bank financial performance, this study recommended decreasing foreign CEOs in Pakistani private commercial banks and giving opportunities to local CEOs. Moreover, this study suggests that Pakistani banks should give opportunities to Pakistani CEOs by conveying their international goals and coursework. Banks suggested increasing their human capital investment by providing conventional education and training. Besides, Pakistani banks suggested considering experience and education when arranging board members and providing more opportunities to the local CEO instead of foreigners.

### Implications

This research has updated regulators and various policymakers with essential bits of knowledge to improve future strategies and performance. This study is more important for Pakistan and for all those countries where there are decisive political and military interventions in the corporate policies; in Pakistan, there is a strong presence of political and military approaches. Next, this study recommends that policymakers further endorse CEO characteristics in Pakistan and other developing countries and limit the political and military interventions in corporate policies, such as India, Bangladesh, other Asian countries, etc.

## Data Availability Statement

The raw data supporting the conclusions of this article will be made available by the authors, without undue reservation.

## Author Contributions

All authors listed have made a substantial, direct and intellectual contribution to the work, and approved it for publication.

## Funding

This paper was funded by the National Social Science Fund of China (Grant No. 16BGL094), Science and Technology, Guangzhou Province, China (Grant No. 2017A040403072), The Innovation Team Project of Guangzhou, China (Grant No. 201831799), and Foundation of Humanities and Social Science Research Program, Ministry of Education (Grant No. 15YJCZH225).

## Conflict of Interest

The authors declare that the research was conducted in the absence of any commercial or financial relationships that could be construed as a potential conflict of interest.

## Publisher's Note

All claims expressed in this article are solely those of the authors and do not necessarily represent those of their affiliated organizations, or those of the publisher, the editors and the reviewers. Any product that may be evaluated in this article, or claim that may be made by its manufacturer, is not guaranteed or endorsed by the publisher.
